# Organic acid production *in vitro *and plant growth promotion in maize under controlled environment by phosphate-solubilizing fluorescent *Pseudomonas*

**DOI:** 10.1186/1471-2180-9-174

**Published:** 2009-08-22

**Authors:** Pratibha Vyas, Arvind Gulati

**Affiliations:** 1 Plant Pathology and Microbiology Laboratory, Institute of Himalayan Bioresource Technology (CSIR), P.O. Box No. 6, Palampur (H.P.)-176 061, India

## Abstract

**Background:**

Phosphorus deficiency is a major constraint to crop production due to rapid binding of the applied phosphorus into fixed forms not available to the plants. Microbial solubilization of inorganic phosphates has been attributed mainly to the production of organic acids. Phosphate-solubilizing microorganisms enhance plant growth under conditions of poor phosphorus availability by solubilizing insoluble phosphates in the soil. This paper describes the production of organic acids during inorganic phosphate solubilization and influence on plant growth as a function of phosphate solubilization by fluorescent *Pseudomonas*.

**Results:**

Nineteen phosphate-solubilizing fluorescent *Pseudomonas *strains of *P. fluorescens*, *P. poae*, *P. trivialis*, and *Pseudomonas *spp. produced gluconic acid, oxalic acid, 2-ketogluconic acid, lactic acid, succinic acid, formic acid, citric acid and malic acid in the culture filtrates during the solubilization of tricalcium phosphate, Mussoorie rock phosphate, Udaipur rock phosphate and North Carolina rock phosphate. The strains differed quantitatively and qualitatively in the production of organic acids during solubilization of phosphate substrates. Cluster analysis based on organic acid profiling revealed inter-species and intra-species variation in organic acids produced by *Pseudomonas *strains. The phosphate-solubilizing bacterial treatments *P. trivialis *BIHB 745, *P. trivialis *BIHB 747, *Pseudomonas *sp. BIHB 756 and *P. poae *BIHB 808 resulted in significantly higher or statistically at par growth and total N, P and K content over single super phosphate treatment in maize. These treatments also significantly affected pH, organic matter, and N, P, and K content of the soil.

**Conclusion:**

The results implied that organic acid production by *Pseudomonas *strains is independent of their genetic relatedness and each strain has its own ability of producing organic acids during the solubilization of inorganic phosphates. Significant difference in plant growth promotion by efficient phosphate-solubilizing *Pseudomonas *strains point at the need for selecting potential strains in plant growth promotion experiments in conjunction with various phosphate substrates for their targeted application as bioinoculants.

## Background

Phosphorus (P) is an essential macronutrient often limiting the plant growth due to its low solubility and fixation in the soil. Improving soil fertility by releasing bound phosphorus by microbial inoculants is an important aspect for increasing crop yield. Phosphorus release from insoluble phosphates reported for several soil microorganisms has been attributed mainly to the production of organic acids and their chelation capacity [1-3]. Direct periplasmic oxidation of glucose to gluconic acid is considered as the metabolic basis of inorganic phosphate solubilization by many Gram-negative bacteria as a competitive strategy to transform the readily available carbon sources into less readily utilizable products by other microorganisms [1,4].

Increased solubilization of fixed soil phosphates and applied phosphates ensuring higher crop yields has been reported on inoculation of phosphate-solubilizing bacteria including *Pseudomonas*, *Bacillus*, *Rhizobium*, *Micrococcus*, *Flavobacterium*, *Burkholderia*, *Achromobacter*, *Erwinia*, and *Agrobacterium *[5,6]. Several *Pseudomonas *species have been reported among the most efficient phosphate-solublizing bacteria and as important bio-inoculants due to their multiple biofertilizing activities of improving soil nutrient status, secretion of plant growth regulators, and suppression of soil-borne pathogens [5,7-9].

Soils in the cold deserts of Lahaul and Spiti in the Indian trans-Himalayan region latitude 31° 44' 57" and 32° 59' 57" North and 76° 46' 29" and 78° 41' 34" East are often with low moisture status, high alkalinity, and low organic matter content. The nutritional problems in such soils are often specific in respect of the low phosphorus availability resulting from their high phosphorus-fixing capacity due to high calcium content [10]. The vast potential of microorganisms for improving productivity in the region remains unexploited [11]. Previously we have reported the isolation, selection, and characterization of stress-tolerant and efficient phosphate-solubilizing fluorescent *Pseudomonas *from the cold deserts of the Himalayas [8,9]. The aim of the present study was to explicate organic acid production during solubilization of inorganic phosphates and effect on plant growth as a function of phosphate solubilization by fluorescent *Pseudomonas*.

## Methods

### Bacterial strains

Nineteen phosphate-solubilizing fluorescent *Pseudomonas *included in the present studies were isolated from the rhizosphere of *Hippophae rhamnoides *growing in the cold deserts of Lahaul and Spiti in the trans-Himalayas and characterized based on their phenotypic characters and 16S rDNA gene sequencing [8,9]. The bacterial strains were maintained at -70°C in nutrient broth supplemented with 20% (v/v) glycerol.

### Production of organic acids during phosphate solubilization

The bacterial strains grown in triplicate in 10 ml NBRIP broth supplemented with 0.5% tricalcium phosphate (TCP), Mussoorie rock phosphate (MRP), Udaipur rock phosphate (URP) and North Carolina rock phosphate (NCRP) at 28°C for 5 days at 180 rpm in a refrigerated incubator shaker (Innova Model 4230, New Brunswick Scientific, USA) were centrifuged at 10,000 rpm for 10 min. and passed through 0.22 μm nylon filter. Quantitative estimation of P-liberated from inorganic phosphates was done using vanado-molybdate method as described earlier [8]. Detection and quantification of organic acids was done on Waters 996 High Performance Liquid Chromatogram (HPLC) equipped with PDA detector, Waters 717 plus autosampler, Waters 600 controller, Waters™ pump, Waters inline degasser AF, and Lichrosphere RP-18 column 250 mm × 4.6 mm and 5 μm particle size (Merck, Germany). The mobile phase was 0.1% ortho-phosphoric acid (Merck, Germany) in the gradient of flow rate as given in Table 1. Eluates were detected at λ 210 nm and identified by retention time and co-chromatography by spiking the sample with the authentic organic acids. The organic acids were quantified by reference to the peak areas obtained for the authentic standards for gluconic acid (Sigma-Aldrich, USA), 2-ketogluconic acid (Sigma, USA), and lactic acid, oxalic acid, malic acid, succinic acid, formic acid, citric acid, malonic acid, propionic acid and tartaric acid (Supelco, USA). Each replicate was analyzed in a single run on HPLC for 76 samples for the four phosphate substrates. The values were presented as the mean of three replicates.

**Table 1:** HPLC elution-profile program.

Time (min)	Flow rate (ml/min)
0–8	0.4
8–14	0.5
14–25	1.2

### Inoculum preparation

The bacterial strains grown in 20 ml trypticase soya broth (TSB) for 48 h at 28°C were centrifuged at 10,000 rpm for 10 min. and the pellets suspended in 0.85% NaCl (OD_600 _= 1.0). The bacterial suspensions were separately mixed with sterilized activated charcoal (4:6 v/w) to give a CFU of approximately 10^7^/g of charcoal-based bacterial inoculants.

### Plant growth under controlled environment

Seeds of *Zea mays *var. Girija surface sterilized with 20% sodium hypochlorite for 3 min. and washed thrice with sterile distilled water were germinated at 25°C in moist sterile vermiculite. Uniformly germinated seeds were coated with the water slurry of charcoal-based microbial inoculants (approx. 5 × 10^5 ^CFU/seed) and two seeds per pot sown in 15 cm diameter pots filled with 2 kg non-sterilized sandy-loam soil. The soil used had pH 6.96, organic matter 3.1%, available N 0.03%, available P 0.0011%, available K 0.013% and available Ca 0.028%. The germinated seeds treated with the water slurry of sterilized activated charcoal without inoculum were used for the control treatments. N and K were applied in the form of ammonium sulfate @ 240 kg N/ha, and muriate of potash @ 80 kg K/ha, respectively. P was applied @ 120 kg P/ha either as single super phosphate (SSP) or tricalcium phosphate (TCP) according to the various treatments. The phosphate-solubilizing bacterial (PSB) treatments included one *P. fluorescens *strain, three *P. poae *strains, ten *P. trivialis *strains, and five *Pseudomonas *spp. strains in combined application with NPK with TCP as the phosphate source. TCP was chosen as phosphate substrate since P-deficiency in soils of the cold deserts of Lahaul and Spiti is attributed mainly to the presence of insoluble di- and tricalcium phosphates. The influence of PSB treatments on plant growth and soil properties was evaluated in comparison to the uninoculated control treatments with or without TCP and SSP. The pots were placed in a complete randomized block design with four replications under 550 μM photon m^-2 ^s^-1 ^mixed incandescent and fluorescent illumination, 16/8 h light/dark cycle and 50–60% RH at 25 ± 2°C in an Environment Control Chamber. The plants were removed carefully under a gentle flow of tap water after 90 days of sowing. Data on root length, plant height (aerial parts), root dry weight and shoot dry weight were recorded. The samples were oven-dried at 70°C for 3 days to a constant weight for determining the dry weight.

### Chemical analyses

The soil samples were air dried and sieved for determining pH, available N, P, K, Ca and organic matter content. The plant samples were oven-dried and powdered for estimation of total N, P and K. Organic matter was determined by the modified Walkley and Black method [12]. Estimation of total N was done by modified Kjeldahl's method, total P by vanado-molybdate yellow colour method, total and available K by flame photometric method, and available Ca in ammonium-acetate extracts [13]. Estimation of available P was estimated by sodium biocarbonate method [14] and available N by alkaline permanganate method [15].

### Experimental design and data analyses

Randomized block design with two factor factorial arrangement was adopted for conducting the experiments. The data were subjected to one-way analysis of variance (ANOVA) and the mean of treatments compared by Duncan's Multiple Range Test at p ≤ 0.01 using SPSS Software version 7.5. Cluster analysis based on the organic acid profiles was performed using STATISTICA data analysis software system version 7 (StatSoft^® ^Inc. Tulsa, USA, 2004).

## Results

### Production of organic acids

**Figure 1:**
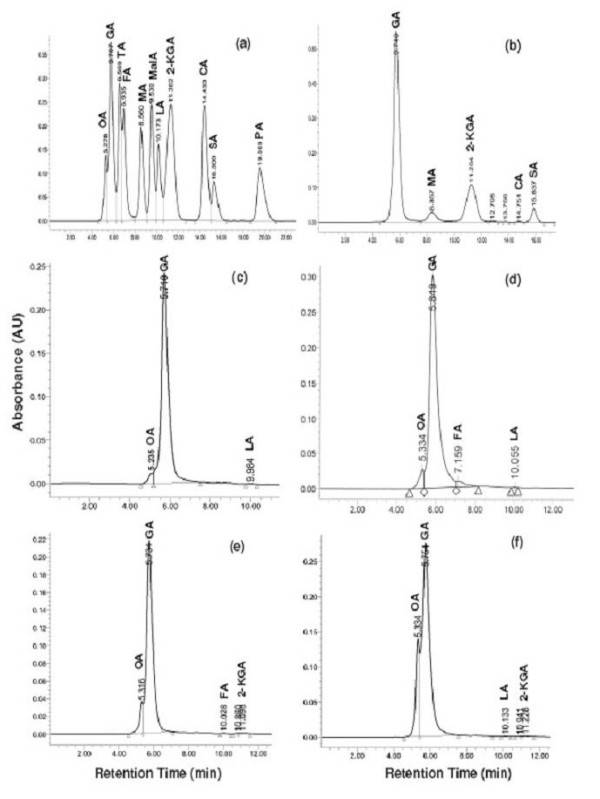
**HPLC chromatograms of authentic organic acids (a) and culture supernatant of *Pseudomonas trivialis *strain BIHB 747 grown for 5 days at 28°C in NBRIP broth with tricalcium phosphate (b), Udaipur rock phosphate (c), Mussoorie rock phosphate (d), North Carolina rock phosphate (e), and North Carolina rock phosphate spiked with OA (f)**. OA = oxalic acid, GA = gluconic acid, TA = tartaric acid, FA = formic acid, MA = malic acid, MalA = malonic acid, LA = lactic acid, 2-KGA = 2-ketogluconic acid, SA = succinic acid, CA = citric acid and PA = propionic acid.

HPLC analysis of the culture filtrates was done to identify and quantity the organic acids produced during the solubilization of TCP, MRP, URP and NCRP by *Pseudomonas fluorescens *strain, three *Pseudomonas poae *strains, ten *Pseudomonas trivialis *strains, and five *Pseudomonas *spp. strains (Fig. 1). During TCP solubilization all strains showed the production of gluconic and 2-ketogluconic acids (Table 2). Apart from one *Pseudomonas *sp. strain no other strain showed oxalic acid production. All strains exhibited the production of malic acid excepting one *Pseudomonas *sp. strain and succinic acid excluding one *Pseudomonas *sp. strain. The production of lactic acid was restricted to one strain of both *P. trivialis *and *Pseudomonas *sp., formic acid to six *P. trivialis*, *P. fluorescens *and two *Pseudomonas *spp. strains, and citric acid to three *P. trivialis *strains and one strain each of *P. poae *and *Pseudomonas *sp., and *P. fluorescens *strain.

**Table 2:** Organic acid production by fluorescent *Pseudomonas *during tricalcium phosphate solubilization.

			Organic acid (μg/ml)								
				
Strain	P-liberated (μg/ml)	Final pH	Oxalic	Gluconic	2-KGA	Lactic	Succinic	Formic	Citric	Malic	Total organic acids (μg/ml)
***P. trivialis***											
BIHB 728	771.3 ± 1.2	3.63	ND	18350.0 ± 5.8	257.0 ± 4.9	49.3 ± 1.8	987.7 ± 3.0	ND	30.5 ± 2.8	2051.8 ± 5.2	21726.3
BIHB 736	778.7 ± 2.4	3.90	ND	18035.3 ± 9.0	177.0 ± 2.6	ND	583.7 ± 4.1	96.0 ± 2.3	ND	1042.0 ± 3.8	19934.0
BIHB 745	827.4 ± 1.8	3.65	ND	18054.3 ± 8.1	210.0 ± 2.9	ND	2249.0 ± 4.4	ND	65.2 ± 2.6	1654.5 ± 3.8	22233.0
BIHB 747	743.0 ± 1.7	3.52	ND	18216.7 ± 3.5	330.7 ± 2.9	ND	1307.7 ± 4.6	ND	25.5 ± 2.1	667.0 ± 3.2	20547.6
BIHB 749	801.0 ± 2.1	3.42	ND	17745.3 ± 7.2	193.7 ± 3.3	ND	797.6 ± 1.9	117.5 ± 2.0	ND	1236.0 ± 6.2	20090.1
BIHB 750	774.3 ± 1.9	3.82	ND	18624.0 ± 4.6	172.3 ± 3.7	ND	509.9 ± 2.7	93.5 ± 1.7	ND	2012.3 ± 3.9	21412.0
BIHB 757	775.3 ± 2.3	3.92	ND	17819.0 ± 6.7	224.5 ± 2.6	ND	772.3 ± 3.4	132.0 ± 3.5	ND	911.0 ± 6.1	19858.8
BIHB 759	751.3 ± 3.7	3.72	ND	18336.3 ± 4.5	179.0 ± 2.9	ND	779.0 ± 5.0	116.0 ± 3.2	ND	2551.0 ± 4.9	21961.3
BIHB 763	718.0 ± 1.5	4.00	ND	17901.3 ± 5.9	173.7 ± 2.6	ND	659.7 ± 4.1	106.0 ± 5.0	ND	2656.0 ± 2.7	21496.7
BIHB 769	806.4 ± 2.3	3.70	ND	19340.0 ± 5.8	154.0 ± 2.5	ND	207.7 ± 3.8	ND	ND	1965.0 ± 5.1	21666.7
***P. poae***											
BIHB 730	768.3 ± 1.8	3.40	ND	17464.7 ± 5.5	251.0 ± 3.1	ND	1172.7 ± 5.9	ND	ND	1718.8 ± 3.4	20607.2
BIHB 752	805.0 ± 1.7	3.50	ND	18800.7 ± 6.4	217.0 ± 4.2	ND	321.3 ± 4.1	ND	ND	3128.0 ± 4.5	22467.0
BIHB 808	821.4 ± 1.7	3.58	ND	18840.3 ± 7.3	176.3 ± 2.3	ND	475.7 ± 6.6	ND	44.3 ± 2.9	75.0 ± 3.6	19611.6
***P. fluorescens***											
BIHB 740	768.3 ± 2.6	3.97	ND	17038.7 ± 3.8	175.3 ± 4.4	ND	163.3 ± 3.5	129.0 ± 3.8	46.0 ± 3.2	3178.0 ± 3.8	20730.3
***Pseudomonas *spp.**											
BIHB 751	318.7 ± 2.0	4.20	7.7 ± 0.6	216.7 ± 3.5	532.3 ± 4.3	ND	ND	23.8 ± 1.7	ND	1181.0 ± 5.9	1961.5
BIHB 756	802.3 ± 2.1	3.53	ND	17937.3 ± 6.2	378.0 ± 3.6	ND	209.4 ± 3.2	ND	ND	4215.0 ± 3.2	22739.7
BIHB 804	805.1 ± 2.2	3.55	ND	17929.7 ± 4.1	122.7 ± 2.4	53.7 ± 1.8	96.0 ± 2.5	ND	ND	1520.0 ± 3.8	19722.1
BIHB 811	717.3 ± 1.9	3.98	ND	14427.3 ± 2.3	14.3 ± 0.4	ND	195.3 ± 4.3	ND	28.5 ± 1.8	ND	14665.4
BIHB 813	631.7 ± 2.5	3.93	ND	18057.7 ± 5.4	175.3 ± 5.9	ND	536.3 ± 4.5	114.4 ± 4.4	ND	913.7 ± 3.7	19797.4

Total organic acids (μg/ml)			7.7	323135.3	4114.1	103.0	12024.3	928.2	240.0	32676.1	373228.7

Values are the mean of three replicates ± standard error of the mean; ND = not detected; 2-KGA = 2-ketogluconic acid.

During URP solubilization the production of oxalic and gluconic acid was detected for all the strains (Table 3). The production of other organic acids was restricted to some strains: 2-ketogluconic acid to three *Pseudomonas *spp. strains and one strain each of *P*. *trivialis*, *P*. *poae *and *P. fluorescens*; lactic acid to five *P. trivialis*, *P. fluorescens *and two *Pseudomonas *spp. strains; succinic acid to one strain each of *P. trivialis*, *P. fluorescens *and *Pseudomonas *sp.; formic acid to two *P. trivialis *strains; and malic acid to four *P. trivialis*, two *P. poae *and four *Pseudomonas *spp. strains. None of the strains showed citric acid production during URP solubilization.

**Table 3:** Organic acid production by fluorescent *Pseudomonas *during Udaipur rock phosphate solubilization.

			Organic acid (μg/ml)								
				
Strain	P-liberated (μg/ml)	Final pH	Oxalic	Gluconic	2-KGA	Lactic	Succinic	Formic	Citric	Malic	Total organic acids (μg/ml)
***P. trivialis***											
BIHB 728	8.7 ± 0.04	3.78	14.3 ± 1.5	6676.7 ± 6.0	ND	52.8 ± 1.3	ND	ND	ND	ND	6743.8
BIHB 736	5.6 ± 0.10	3.79	10.6 ± 1.5	7116.0 ± 5.9	ND	ND	ND	ND	ND	ND	7126.6
BIHB 745	8.3 ± 0.30	3.78	11.1 ± 0.9	8190.0 ± 5.8	ND	ND	ND	35.1 ± 3.1	ND	53.4 ± 3.7	8289.6
BIHB 747	4.4 ± 0.01	3.71	10.3 ± 1.1	6962.3 ± 5.0	ND	41.3 ± 2.0	ND	ND	ND	ND	7013.9
BIHB 749	5.3 ± 0.01	3.60	11.4 ± 0.7	7921.7 ± 6.9	ND	41.3 ± 3.5	ND	ND	ND	ND	7974.4
BIHB 750	6.1 ± 0.02	3.87	9.4 ± 0.8	7496.7 ± 6.0	ND	ND	ND	ND	ND	34.5 ± 2.5	7540.6
BIHB 757	7.1 ± 0.04	3.72	8.7 ± 1.2	5459.0 ± 3.1	ND	ND	ND	ND	ND	ND	5467.7
BIHB 759	14.0 ± 0.90	3.62	9.5 ± 1.0	6850.0 ± 6.4	ND	ND	ND	ND	ND	ND	6859.5
BIHB 763	9.3 ± 0.04	3.78	26.6 ± 0.7	10903.0 ± 3.6	ND	42.8 ± 1.0	ND	93.6 ± 2.0	ND	103.6 ± 3.3	11169.6
BIHB 769	7.6 ± 0.50	3.70	12.4 ± 1.5	2964.0 ± 3.1	20.5 ± 2.3	92.3 ± 1.8	56.1 ± 4.6	ND	ND	383.0 ± 3.1	3528.3
***P. poae***											
BIHB 730	5.0 ± 0.09	3.70	25.7 ± 1.4	5055.3 ± 5.0	16.4 ± 1.2	ND	ND	ND	ND	ND	5097.4
BIHB 752	7.7 ± 0.10	3.90	8.0 ± 0.8	7119.0 ± 3.8	ND	ND	ND	ND	ND	35.5 ± 3.4	7162.5
BIHB 808	7.6 ± 0.05	3.83	9.5 ± 1.3	7616.3 ± 3.5	ND	ND	ND	ND	ND	36.3 ± 3.3	7662.1
***P. fluorescens***											
BIHB 740	3.8 ± 0.05	4.00	12.7 ± 1.0	1117.7 ± 5.4	67.0 ± 2.6	164.0 ± 2.6	102.3 ± 1.5	ND	ND	ND	1463.7
***Pseudomonas *spp.**											
BIHB 751	1.4 ± 0.03	4.20	13.9 ± 0.8	631.7 ± 4.4	255.0 ± 5.1	ND	ND	ND	ND	4350.0 ± 2.5	5250.6
BIHB 756	9.4 ± 0.05	3.75	11.9 ± 0.8	5061.7 ± 9.4	51.7 ± 2.5	ND	ND	ND	ND	57.7 ± 2.7	5183.0
BIHB 804	3.8 ± 0.40	4.03	12.5 ± 0.9	5839.3 ± 7.8	ND	43.2 ± 2.0	ND	ND	ND	41.8 ± 2.5	5936.8
BIHB 811	6.1 ± 0.05	4.11	17.1 ± 1.2	4412.3 ± 5.2	138.8 ± 0.9	121.3 ± 1.5	108.0 ± 3.1	ND	ND	658.1 ± 2.3	5455.6
BIHB 813	5.2 ± 0.30	4.32	12.0 ± 1.5	5971.7 ± 5.2	ND	ND	ND	ND	ND	ND	5983.7

Total organic acids (μg/ml)			235.6	97392.7	549.4	599	266.4	128.7	0	5753.9	104925.7

Values are the mean of three replicates ± standard error of the mean; ND = not detected; 2-KGA = 2-ketogluconic acid.

During MRP solubilization the production of oxalic and gluconic acid was also detected for all the strains (Table 4). The production of 2-ketogluconic acid was shown by one *Pseudomonas poae*, *P. fluorescens *and four *Pseudomonas *spp. strains, lactic acid by five *P. trivialis*, one *P. poae *and three *Pseudomonas *spp. strains, succinic acid by three *Pseudomonas *spp. strains, formic acid by three *P. trivialis *and three *Pseudomonas *spp. strains, formic acid by *P. fluorescens *and three *P. trivialis *strains, malic acid by two *P. trivialis*, one *P. poae*, *P. fluorescens *and four *Pseudomonas *spp. strains, and citric acid by one *Pseudomonas *sp. strain.

**Table 4:** Organic acid production by fluorescent *Pseudomonas *during Mussoorie rock phosphate solubilization.

			Organic acid (μg/ml)								
				
Strain	P-liberated (μg/ml)	Final pH	Oxalic	Gluconic	2-KGA	Lactic	Succinic	Formic	Citric	Malic	Total organic acids (μg/ml)
***P. trivialis***											
BIHB 728	11.0 ± 0.3	3.52	15.1 ± 1.4	8443.3 ± 6.0	ND	44.9 ± 1.7	ND	ND	ND	ND	8503.3
BIHB 736	13.1 ± 0.1	3.52	15.6 ± 1.4	9314.3 ± 7.4	ND	ND	ND	ND	ND	ND	9329.9
BIHB 745	5.8 ± 0.3	3.63	14.8 ± 1.4	9394.0 ± 8.3	ND	ND	ND	84.0 ± 3.1	ND	930.0 ± 4.2	10422.8
BIHB 747	12.0 ± 0.2	3.49	16.3 ± 0.7	10016.7 ± 4.4	ND	36.8 ± 2.0	ND	70.4 ± 2.7	ND	ND	10140.2
BIHB 749	8.0 ± 0.04	3.59	15.8 ± 0.7	12027.0 ± 5.7	ND	ND	ND	ND	ND	ND	12042.8
BIHB 750	4.8 ± 0.4	3.67	11.7 ± 0.9	8460.0 ± 5.8	ND	ND	ND	ND	ND	32.3 ± 2.1	8504.0
BIHB 757	9.0 ± 0.04	3.63	10.6 ± 1.0	9460.0 ± 5.5	ND	39.4 ± 2.8	ND	ND	ND	ND	9510.0
BIHB 759	11.0 ± 0.2	3.52	16.7 ± 1.3	13854.0 ± 4.9	ND	39.7 ± 1.3	ND	ND	ND	ND	13910.4
BIHB 763	12.9 ± 0.02	3.50	18.2 ± 0.5	13444.0 ± 5.5	ND	ND	ND	87.7 ± 3.0	ND	ND	13549.9
BIHB 769	6.1 ± 0.4	3.65	16.4 ± 0.7	11633.7 ± 5.4	ND	40.5 ± 2.6	ND	ND	ND	ND	11690.6
***P. poae***											
BIHB 730	4.0 ± 0.06	4.62	12.5 ± 1.3	7871.0 ± 8.5	19.9 ± 1.4	37.8 ± 2.1	ND	ND	ND	ND	7941.2
BIHB 752	6.0 ± 0.03	3.62	19.6 ± 2.1	15727.0 ± 5.9	ND	ND	ND	ND	ND	293.0 ± 4.7	16039.6
BIHB 808	8.6 ± 0.6	3.53	15.3 ± 1.2	13749.7 ± 3.4	ND	ND	ND	ND	ND	ND	13765.0
***P. fluorescens***											
BIHB 740	3.0 ± 0.1	5.90	14.3 ± 0.9	8051.0 ± 6.1	468.0 ± 3.1	ND	ND	114.4 ± 4.9	ND	183.2 ± 4.9	8830.9
***Pseudomonas *spp.**											
BIHB 751	2.4 ± 0.1	3.89	11.7 ± 0.4	7076.3 ± 4.6	126.3 ± 7.2	ND	ND	ND	ND	2802.0 ± 4.7	10016.3
BIHB 756	12.7 ± 0.4	3.53	14.7 ± 1.2	9120.0 ± 6.4	153.0 ± 3.1	ND	142.0 ± 3.5	ND	ND	264.0 ± 4.6	9693.7
BIHB 804	8.1 ± 0.3	3.55	39.3 ± 1.5	8997.0 ± 7.2	18.4 ± 0.9	39.6 ± 1.1	ND	ND	ND	34.1 ± 2.9	9128.4
BIHB 811	2.9 ± 0.03	4.00	42.0 ± 1.7	10007.0 ± 3.8	234.3 ± 2.0	50.8 ± 2.3	349.7 ± 2.7	ND	22.3 ± 2.2	36.1 ± 2.8	10742.2
BIHB 813	2.2 ± 0.4	4.05	14.2 ± 0.7	10396.0 ± 5.6	ND	40.5 ± 2.0	136.0 ± 2.1	ND	ND	ND	10586.7

Total organic acids (μg/ml)			334.8	197042.0	1019.9	370.0	627.7	356.5	22.3	4574.7	204347.9

Values are the mean of three replicates ± standard error of the mean; ND = Not detected; 2-KGA = 2-ketogluconic acid.

In NCRP solubilization the production of oxalic acid and gluconic acid was detected for all the strains (Table 5). The production of other organic acids was limited to some strains: 2-ketogluconic acid to five *P. trivialis*, two *P. poae*, *P. fluorescens *and three *Pseudomonas *spp. strains; lactic acid to three *P. trivialis *and four *Pseudomonas *spp. strains; succinic acid to one strain each of *P. poae*, *P. fluorescens *and *Pseudomonas *sp.; formic acid to *P. fluorescens *strain; citric acid to one strain each of *P. poae *and *Pseudomonas *sp.; and malic acid to one *P. trivialis*, *P. fluorescens *and three *Pseudomonas *spp. strains.

**Table 5:** Organic acid production by fluorescent *Pseudomonas *during North Carolina rock phosphate solubilization.

			Organic acid (μg/ml)								
				
Strain	P-liberated (μg/ml)	Final pH	Oxalic	Gluconic	2-KGA	Lactic	Succinic	Formic	Citric	Malic	Total organic acids (μg/ml)
***P. trivialis***											
BIHB 728	191.3 ± 1.0	3.70	14.7 ± 0.6	3810.0 ± 7.6	10.2 ± 1.0	ND	ND	ND	ND	ND	3834.9
BIHB 736	172.0 ± 0.3	3.72	9.1 ± 1.3	4672.3 ± 6.4	ND	42.7 ± 1.2	ND	ND	ND	ND	4724.1
BIHB 745	168.2 ± 0.4	3.73	10.8 ± 0.5	3880.7 ± 5.2	10.1 ± 0.8	ND	ND	ND	ND	ND	3901.6
BIHB 747	173.0 ± 0.4	3.81	16.6 ± 1.0	6035.0 ± 4.2	11.0 ± 1.8	40.3 ± 2.9	ND	ND	ND	ND	6102.9
BIHB 749	177.3 ± 0.6	3.73	17.1 ± 0.9	4587.0 ± 4.7	ND	42.7 ± 2.2	ND	ND	ND	113.2 ± 2.7	4760.0
BIHB 750	145.7 ± 1.2	3.88	10.3 ± 0.6	4395.3 ± 7.7	ND	ND	ND	ND	ND	ND	4405.6
BIHB 757	175.0 ± 0.3	3.92	13.6 ± 2.3	4649.0 ± 5.5	13.3 ± 1.1	ND	ND	ND	ND	ND	4675.9
BIHB 759	178.0 ± 0.6	3.81	11.0 ± 1.4	5331.0 ± 6.1	ND	ND	ND	ND	ND	ND	5342.0
BIHB 763	161.2 ± 0.2	3.80	11.5 ± 1.3	4362.0 ± 4.6	10.8 ± 1.0	ND	ND	ND	ND	ND	4384.3
BIHB 769	224.0 ± 0.7	3.55	10.8 ± 0.8	4448.0 ± 5.3	ND	ND	ND	ND	ND	ND	4458.8
***P. poae***											
BIHB 730	163.8 ± 1.1	3.90	10.1 ± 1.2	3770.0 ± 6.4	ND	ND	ND	ND	ND	ND	3780.1
BIHB 752	204.3 ± 0.7	3.72	12.7 ± 1.5	4947.0 ± 6.0	10.3 ± 1.0	ND	ND	ND	26.1 ± 2.0	ND	4996.1
BIHB 808	193.4 ± 0.7	3.65	11.5 ± 1.2	4420.3 ± 2.9	10.9 ± 0.8	ND	45.1 ± 4.3	ND	ND	ND	4442.7
***P. fluorescens***											
BIHB 740	236.8 ± 0.6	3.48	9.8 ± 1.1	4762.7 ± 4.3	31.3 ± 2.0	ND	46.7 ± 3.2	59.3 ± 3.5	ND	104.8 ± 3.0	5014.6
***Pseudomonas *spp.**											
BIHB 751	123.3 ± 1.4	3.89	9.1 ± 1.1	3241.0 ± 2.6	22.3 ± 1.9	ND	ND	ND	ND	415.0 ± 4.0	3687.4
BIHB 756	164.2 ± 0.8	3.82	11.3 ± 0.6	4975.0 ± 7.5	ND	41.7 ± 1.4	ND	ND	29.5 ± 2.2	ND	5057.5
BIHB 804	161.5 ± 1.0	3.78	15.7 ± 1.2	4542.0 ± 5.3	10.5 ± 1.0	39.3 ± 2.0	ND	ND	ND	33.0 ± 1.2	4640.5
BIHB 811	173.0 ± 1.1	3.92	15.5 ± 0.8	2549.0 ± 5.9	32.7 ± 0.9	54.3 ± 2.0	75.1 ± 4.6	ND	ND	265.0 ± 3.6	2991.6
BIHB 813	92.7 ± 1.2	4.07	8.9 ± 1.2	4633.3 ± 5.5	ND	38.8 ± 2.0	ND	ND	ND	ND	4681.0

Total organic acids (μg/ml)			230.1	84010.6	173.4	299.8	121.8	59.3	55.6	931	85881.6

Values are the mean of three replicates ± standard error of the mean; ND = Not detected; 2-KGA = 2-ketogluconic acid.

Quantitative difference in the production of organic acids was observed during the solubilization of phosphate substrates by *Pseudomonas *strains (Tables 2, 3, 4, 5). The quantities of organic acids produced during TCP solubilization ranged from 216.7–19340 μg/ml gluconic acid, 14.3–532.3 μg/ml 2-ketogluconic acid, 96–2249 μg/ml succinic acid, 23.8–132.0 μg/ml formic acid, 25.5–65.2 μg/ml citric acid, and 75–4215 μg/ml malic acid. Lactic acid production shown only by *P. trivialis *BIHB 728 and *Pseudomonas *sp. BIHB 804 was 53.7 and 49.3 μg/ml, respectively. Oxalic acid production detected only for *Pseudomonas *sp. BIHB 751 was 318.7 μg/ml during TCP solubilization. Organic acid production during URP solubilization varied from 8–26.6 μg/ml oxalic acid, 631.7–10903 μg/ml gluconic acid, 16.4–255 μg/ml 2-ketogluconic acid, 41.3–164 μg/ml lactic acid, 56.1–108 μg/ml succinic acid, and 34.5–4350 μg/ml malic acid. Formic acid production only by *P. trivialis *BIHB 745 and *P. trivialis *BIHB 763 was 35.1 and 93.6 μg/ml, respectively. During MRP solubilization the quantities of organic acids estimated in the culture filtrates were 10.6–39.3 μg/ml oxalic acid, 7076.3–15727 μg/ml gluconic acid, 18.4–468 μg/ml 2-ketogluconic acid, 36.8–50.8 μg/ml lactic acid, 136.0–349.7 μg/ml succinic acid, 70.4–114.4 μg/ml formic acid, and 32.3–2802 μg/ml malic acid. Citric acid production observed for only *Pseudomonas *sp. BIHB 811 was 22.3 μg/ml during MRP solubilization. Organic acids during NCRP solubilization ranged from 8.9–17.1 μg/ml oxalic acid, 2549–6035 μg/ml gluconic acid, 10.1–32.7 μg/ml 2-ketogluconic acid, 38.8–54.3 μg/ml lactic acid, 45.1–75.1 μg/ml succinic acid, and 33–415 μg/ml malic acid. Citric acid production shown by the two strains *P. poae *BIHB 752 and *Pseudomonas *sp. BIHB 756 was 26.1 and 29.5 μg/ml, respectively. *Pseudomonas fluorescens *BIHB 740 produced 59.3 μg/ml formic acid during NCRP solubilization.

**Figure 2:**
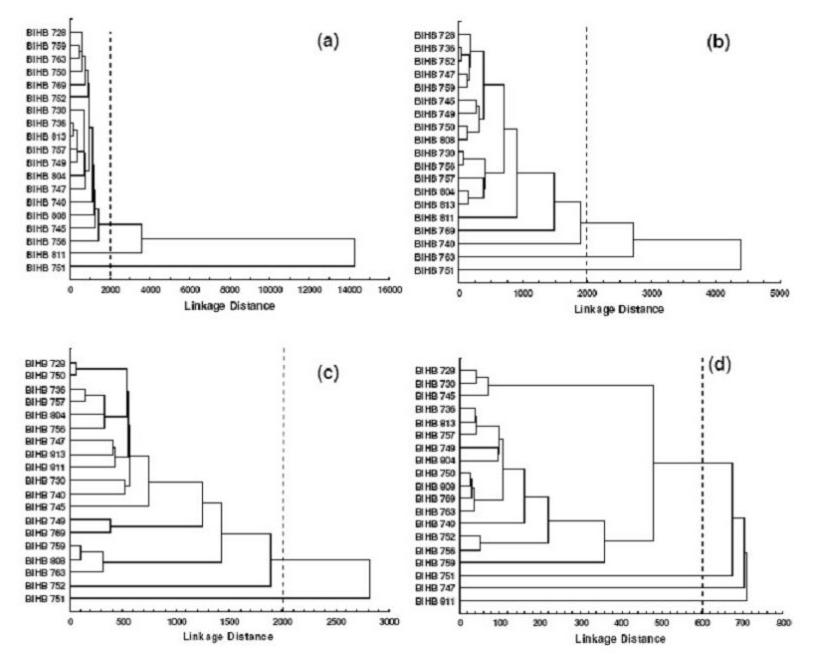
**Dendrogram based on organic acid profiles of phosphate-solubilizing fluorescent *Pseudomonas *grown in NBRIP broth with (a) tricalcium phosphate, (b) Udaipur rock phosphate, (c) Mussoorie rock phosphate, and (d) North Carolina rock phosphate after 5 days incubation at 28°C**.

Cluster analysis based on the organic acid profiles during TCP, URP, MRP and NCRP solubilization generated *Pseudomonas *groups with strains belonging to the same or different species (Fig. 2). For TCP solubilization a single cluster was obtained at 2000 linkage distance, while *Pseudomonas *sp. BIHB 751 and *Pseudomonas *sp. BIHB 811 stood outside the cluster (Fig. 2a). *Pseudomonas *sp. BIHB 751 differed from the other strains in producing oxalic acid, lack of succinic acid production, and producing the lowest quantity of gluconic acid and the highest quantity of 2-ketogluconic acid. *Pseudomonas *sp. BIHB 811 showed dissimilarity in not producing malic acid. In URP solubilization a single cluster of three sub-clusters and single branches of *Pseudomonas *sp. BIHB 811, *P. trivialis *BIHB 769 and *P. fluorescens *BIHB 740 were formed at 2000 linkage distance, while *Pseudomonas *sp. BIHB 751 and *P. trivialis *BIHB 763 stood independently outside the cluster (Fig. 2b). *Pseudomonas *sp. BIHB 751 differed in producing the lowest quantity of gluconic acid and the highest quantities of 2-ketogluconic and malic acids. *Pseudomonas trivialis *BIHB 763 was separate from other strains in producing the highest quantities of gluconic and formic acids (Fig. 2b). During MRP solubilization a single cluster including six sub-clusters and two single branches of *P. trivialis *BIHB 745 and *P. poae *BIHB 752 were observed at 2000 linkage distance. *Pseudomonas *sp. BIHB 751 stood separately outside the cluster in producing the lowest quantity of gluconic acid and the highest quantity of malic acid (Fig. 2c). In NCRP solubilization *P. trivialis *BIHB 747, *Pseudomonas *sp. BIHB 751 and *Pseudomonas *sp. BIHB 811 stood outside the cluster as independent branches at 600 linkage distance (Fig 2d). The cluster incorporated 5 sub-clusters and separate branches of *Pseudomonas *sp. BIHB 740 and *P. trivialis *BIHB 759. *Pseudomonas trivialis *BIHB 747 differed in the highest gluconic acid production, *Pseudomonas *sp. BIHB 751 in the highest malic acid production, and *Pseudomonas *sp. BIHB 811 in producing the lowest quantity of gluconic acid and the highest quantity of 2-ketogluconic, lactic, and succinic acids.

### Influence on plant growth

Significant difference was observed for the growth parameters in maize among PSB treatments and uninoculated control treatments (Table 6). The plant height was significantly higher in fifteen PSB treatments and NP_SSP_K over NP_0_K. Increment in height was significantly higher with NP_TCP_K+*Pt *BIHB 759 and significantly lower with NP_TCP_K+*P*sp BIHB 751 over NP_SSP_K. Other PSB treatments were statistically at par with NP_SSP_K. Nine PSB treatments exhibited significantly higher plant height over NP_TCP_K. Plant height increase was the highest with NP_TCP_K+*Pt *BIHB 759, followed by NP_TCP_K+*Pt *BIHB 763, NP_TCP_K+*Pt *BIHB 745, NP_TCP_K+*Pp *BIHB 808, NP_TCP_K+*Pt *BIHB 757, and NP_TCP_K+*Pt *BIHB 747 treatments.

**Table 6:** Influence of fluorescent *Pseudomonas *on growth and nutrient content of maize after 90 days in Environment Control Chamber.

	Growth parameter				Nutrient content (%)					
	
					Shoot			Root		
					
Treatment	Plant height (cm)	Shoot DW (g/plant)	Root length (cm)	Root DW (g/plant)	N	P	K	N	P	K
NP_0_K	116.1^h^	4.03^f^	17.5^g^	0.47^hi^	1.83^d^	0.18^j^	2.50^ef^	1.39^g^	0.08^i^	0.61^d^
NP_TCP_K	126.4^fgh^	4.38^ef^	18.5^fg^	0.55^hi^	1.95^cd^	0.24^ij^	2.37^f^	1.40^fg^	0.14^hi^	0.65^cd^
NP_SSP_K	135.5^bcdef^	4.61^ef^	20.3^efg^	0.88^de^	1.98^cd^	0.31^hij^	2.63^cdef^	1.43^efg^	0.25^defg^	0.70^cd^
NP_TCP_K+*Pt *BIHB 728	131.1^cdefg^	^4.84^ef	20.9^defg^	0.64^gh^	1.95^cd^	0.37^efghi^	2.67^cdef^	1.97^ab^	0.26^defg^	0.93^ab^
NP_TCP_K+*Pt *BIHB 736	130.0^efg^	4.51^ef^	27.1^a^	0.55^hi^	2.22^abcd^	0.34^ghi^	3.13^abcde^	2.03^a^	0.21^gh^	0.85^abc^
NP_TCP_K+*Pt *BIHB 745	145.9^ab^	7.57^abc^	26.6^ab^	1.16^b^	2.72^ab^	0.64^a^	3.43^ab^	1.91^abc^	0.40^a^	0.98^a^
NP_TCP_K+*Pt *BIHB 747	142.0^abcde^	7.79^ab^	24.8^abcd^	1.11^bc^	2.63^abc^	0.56^abc^	3.10^abcde^	1.84^abcde^	0.32^bcde^	0.86^abc^
NP_TCP_K+*Pt *BIHB 749	141.5^abcde^	6.04^bcde^	24.9^abcd^	1.34^a^	2.20^abcd^	0.43^cdefgh^	2.92^bcdef^	1.50^cdefg^	0.23^fg^	0.74^bcd^
NP_TCP_K+*Pt *BIHB 750	126.8^fgh^	4.75^ef^	20.9^defg^	0.51^hi^	2.18^abcd^	0.57^abc^	2.60^def^	1.55^bcdefg^	0.31^cde^	0.74^bcd^
NP_TCP_K+*Pt *BIHB 757	142.6^abcd^	5.63^def^	23.5^abcd^	1.08^bc^	2.45^abcd^	0.50^abcdef^	2.83^bcdef^	1.63^abcdefg^	0.24^efg^	0.79^abcd^
NP_TCP_K+*Pt *BIHB 759	148.8^a^	5.14^def^	25.8^abc^	0.62^gh^	2.49^abcd^	0.53^abcd^	3.47^ab^	1.93^ab^	0.30^cdef^	0.74^bcd^
NP_TCP_K+*Pt *BIHB 763	146.0^ab^	4.82^ef^	24.0^abcd^	0.66^fgh^	2.60^abc^	0.49^bcdefg^	2.93^bcdef^	1.70^abcdefg^	0.26^defg^	0.83^abcd^
NP_TCP_K+*Pt *BIHB 769	141.0^abcde^	7.70^abc^	26.5^ab^	0.84^def^	2.10^bcd^	0.39^defgh^	2.60^def^	1.56^bcdefg^	0.23^fg^	0.74^bcd^
NP_TCP_K+*Pp *BIHB 730	126.4^fgh^	^8.55^a	26.5^ab^	0.81^efg^	2.27^abcd^	0.51^abcde^	2.77^cdef^	1.49^cdefg^	0.25^defg^	0.74^bcd^
NP_TCP_K+*Pp *BIHB 752	130.6^defg^	5.89^cdef^	22.4^bcdef^	0.52^hi^	2.15^bcd^	0.36^fghi^	3.27^abc^	1.95^ab^	0.39^ab^	0.78^abcd^
NP_TCP_K+*Pp *BIHB 808	143.5^abc^	5.46^def^	24.1^abcd^	0.63^gh^	2.64^abc^	0.63^ab^	3.10^abcde^	1.88^abcd^	0.27^cdef^	0.68^cdcd^
NP_TCP_K+*Pf *BIHB 740	137.0^abcdefg^	6.83^abcd^	24.8^abcd^	1.01^bcd^	2.58^abc^	0.39^defgh^	2.75^cdef^	1.43^efg^	0.24^defg^	0.82^abcd^
NP_TCP_K+*P*sp BIHB 751	119.5^gh^	4.84^ef^	22.5^bcdef^	0.41^i^	2.58^abc^	0.30^hij^	2.72^cdef^	1.47^defg^	0.20^gh^	0.62^d^
NP_TCP_K+*P*sp BIHB 756	141.1^abcde^	6.88^abcd^	26.0^ab^	0.92^cde^	2.88^a^	0.61^ab^	3.67^a^	1.90^abc^	0.35^abc^	0.82^abcd^
NP_TCP_K+*P*sp BIHB 804	131.4^cdefg^	5.03^def^	23.4^abcd^	0.96^cde^	2.40^abcd^	0.59^ab^	3.17^abcd^	1.37^g^	0.20^gh^	0.79^abcd^
NP_TCP_K+*P*sp BIHB 811	127.3^fgh^	4.46^ef^	18.5^fg^	0.58^hi^	2.25^abcd^	0.31^hij^	2.63^cdef^	1.95^ab^	0.32^bcd^	0.77^bcd^
NP_TCP_K+*P*sp BIHB 813	130.9^defg^	8.58^a^	21.4^cdefg^	0.48^hi^	2.47^abcd^	0.39^defgh^	3.27^abc^	1.82^abcdefg^	0.22^gh^	0.76^bcd^

Values are the mean of 8 replicates. N and K applied as ammonium sulfate @ 240 kg N/ha, and muriate of potash @ 80 kg K/ha to all the treatments, respectively. TCP = tricalcium phosphate (120 kg P/ha). SSP = single super phosphate (120 kg P/ha). Values with common letters in each column do not differ statistically according to Duncan's Multiple Range Test at p ≤ 0.01. DW = dry weight, *Pt = P. trivialis*, *Pp *= *P. poae*, *Pf *= *P. fluorescens*, and *P*sp = *Pseudomonas*

The shoot dry weight was significantly higher in seven PSB treatments over NP_0_K, NP_TCP_K and NP_SSP_K. The highest shoot dry weight with NP_TCP_K+*P*sp BIHB 813 was statistically at par with NP_TCP_K+*Pp *BIHB 730, NP_TCP_K+*Pt *BIHB 747, NP_TCP_K+*Pt *BIHB 769, NP_TCP_K+*Pt *BIHB 745, NP_TCP_K+*P*sp BIHB 756 and NP_TCP_K+*Pf *BIHB 740. The root length was significantly higher in fifteen PSB treatments over NP_0_K and thirteen PSB treatments over NP_TCP_K and NP_SSP_K. The maximum increase was obtained with NP_TCP_K+*Pt *BIHB 736, followed by NP_TCP_K+*Pt *BIHB 745, NP_TCP_K+*Pt *BIHB 769, NP_TCP_K+*Pp *BIHB 730 and NP_TCP_K+*P*sp BIHB 756. The treatments NP_TCP_K and NP_SSP_K were statistically at par with NP_0_K. The root dry weight was significantly higher in NP_TCP_K+*Pt *BIHB 749 over other PSB treatments, NP_0_K, NP_TCP_K and NP_SSP_K. The treatments NP_TCP_K+*Pt *BIHB 745, NP_TCP_K+*Pt *BIHB 747 and NP_TCP_K+*Pt *BIHB 757 were statistically at par and showed significantly higher root dry weight over NP_0_K, NP_TCP_K and NP_SSP_K.

### Plant NPK content

The treatments showed significant difference in the nutrient content of roots and shoots (Table 6). The shoot N was statistically higher in seven PSB treatments over NP_0_K and two PSB treatments over NP_0_K, NP_TCP_K and NP_SSP_K. A non-significant difference in the shoot N was observed with NP_0_K, NP_TCP_K and NP_SSP_K. The shoot P was significantly higher in ten PSB treatments over NP_0_K, NP_TCP_K and NP_SSP_K. The highest P content obtained with NP_TCP_K+*Pt *BIHB 745. The treatments NP_TCP_K and NP_SSP_K were statistically at par with NP_0_K. The shoot K was significantly higher in NP_TCP_K+*Psp *BIHB 756, NP_TCP_K+*Pt *BIHB 759 and NP_TCP_K+*Pt *BIHB 745 over NP_0_K, NP_TCP_K and NP_SSP_K.

The root N was significantly higher in eight PSB treatments over NP_0_K, NP_TCP_K and NP_SSP_K. The N content was statistically at par in NP_0_K, NP_TCP_K and NP_SSP_K. The highest N was obtained with NP_TCP_K+*Pt *BIHB 736. The root P was significantly higher in three PSB treatments over NP_SSP_K. The maximum increase was obtained with NP_TCP_K+*Pt *BIHB 745, followed by NP_TCP_K+*Pp *BIHB 752 and NP_TCP_K+*P*sp BIHB 756. The P content was significantly higher in NP_SSP_K over NP_0_K and NP_TCP_K. The root K was significantly higher in NP_TCP_K+*Pt *BIHB 745 and NP_TCP_K+*Pt *BIHB 728 over NP_0_K, NP_TCP_K and NP_SSP_K. Other treatments were statistically at par with NP_TCP_K and NP_SSP_K.

### Soil properties

The soil pH, organic matter and available N, P, K contents were significantly affected by PSB treatments (Table 7). The final pH with non-significant difference among various treatments was less than the initial pH. The highest decrease recorded with NP_TCP_K+*Pt *BIHB 757 was statistically at par with all other PSB treatments but significantly lower than NP_0_K, NP_TCP_K and NP_SSP_K. The organic matter content was significantly higher in four PSB treatments than NP_0_K, NP_TCP_K and NP_SSP_K.

**Table 7:** Influence of fluorescent *Pseudomonas *on soil properties after 90 days in maize in Environment Control Chamber.

			Available nutrients (%)			
			
Treatment	pH	OM (%)	N	P	K	Ca
NP_0_K	6.73^a^	3.40^ghi^	0.044^hij^	0.0015^kl^	0.020^fgh^	0.032^i^
NP_TCP_K	6.63^ab^	3.63^defghi^	0.049^efgh^	0.0021^ghij^	0.025^cde^	0.038^h^
NP_SSP_K	6.50^abc^	3.48^efghi^	0.046^fghi^	0.0025^defg^	0.022^efg^	0.033^hi^
NP_TCP_K+*Pt *BIHB 728	6.26^abcd^	3.90^bcde^	0.052^def^	0.0019^ijkl^	0.025^cde^	0.069^bc^
NP_TCP_K+*Pt *BIHB 736	6.23^bcd^	3.42^fghi^	0.057^bcd^	0.0026^defg^	0.024^def^	0.057^fg^
NP_TCP_K+*Pt *BIHB 745	5.93^d^	4.17^ab^	0.065^a^	0.0038^a^	0.033^ab^	0.085^a^
NP_TCP_K+*Pt *BIHB 747	6.02^cd^	4.13^abc^	0.062^ab^	0.0027^cdef^	0.030^abc^	0.081^a^
NP_TCP_K+*Pt *BIHB 749	6.12^cd^	3.57^efghi^	0.042^ijk^	0.0024^efgh^	0.029^bc^	0.074^b^
NP_TCP_K+*Pt *BIHB 750	6.24^bcd^	3.55^efghi^	0.039^jkl^	0.0019^ijkl^	0.019^fgh^	0.080^a^
NP_TCP_K+*Pt *BIHB 757	5.93^d^	3.79^bcdefg^	0.059^bc^	0.0024^efgh^	0.026^cde^	0.070^bc^
NP_TCP_K+*Pt *BIHB 759	6.20^bcd^	4.00^abcd^	0.040^jk^	0.0022^fghi^	0.022^efgh^	0.072^b^
NP_TCP_K+*Pt *BIHB 763	6.18^bcd^	3.82^bcdefg^	0.039^kl^	0.0028^cde^	0.018^gh^	0.058^ef^
NP_TCP_K+*Pt *BIHB 769	6.30^abcd^	3.29^i^	0.046^ghi^	0.0026^cdef^	0.027^cde^	0.059^e^
NP_TCP_K+*Pp *BIHB 730	6.23^bcd^	3.55^efghi^	0.050^efg^	0.0020^hijkl^	0.027^cde^	0.052^g^
NP_TCP_K+*Pp *BIHB 752	6.17^bcd^	3.89^bcde^	0.037^kl^	0.0020^hijk^	0.018^gh^	0.057^fg^
NP_TCP_K+*Pp *BIHB 808	6.21^bcd^	3.43^fghi^	0.049^fgh^	0.0017^ijkl^	0.022^efg^	0.061^de^
NP_TCP_K+*Pf *BIHB 740	6.25^bcd^	3.85^bcdef^	0.055^cde^	0.0021^ghij^	0.027^cde^	0.072^b^
NP_TCP_K+*P*sp BIHB 751	6.33^abcd^	3.43^fghi^	0.034^l^	0.0016^jkl^	0.017^h^	0.053^fg^
NP_TCP_K+*P*sp BIHB 756	6.13^bcd^	4.32^a^	0.060^abc^	0.0033^b^	0.035^a^	0.072^b^
NP_TCP_K+*P*sp BIHB 804	6.18^bcd^	3.74^cdefgh^	0.049^efgh^	0.0015^l^	0.028^bcd^	0.069^bc^
NP_TCP_K+*P*sp BIHB 811	6.19^bcd^	4.06^abc^	0.051^efg^	0.0031^bc^	0.022^efg^	0.062^de^
NP_TCP_K+*P*sp BIHB 813	6.17^bcd^	3.36^hi^	0.049^fgh^	0.0030^bcd^	0.025^cde^	0.065^cd^

Values are the mean of 8 replicates. N and K applied as ammonium sulfate @ 240 kg N/ha, and muriate of potash @ 80 kg K/ha to all the treatments, respectively. TCP = tricalcium phosphate (120 kg P/ha). SSP = single super phosphate (120 kg P/ha). Values with common letters in each column do not differ statistically according to Duncan's Multiple Range Test at p ≤ 0.01. *Pt = P. trivialis*, *Pp *= *P. poae*, *Pf *= *P. fluorescens*, and *P*sp = *Pseudomonas *sp.

The soil N content was significantly higher in five PSB treatments than NP_0_K, NP_TCP_K and NP_SSP_K and statistically at par among NP_0_K, NP_TCP_K and NP_SSP_K. The soil P content was significantly higher in three PSB treatments over NP_0_K, NP_TCP_K and NP_SSP_K. The highest available P content was obtained with NP_TCP_K+*Pt *BIHB745 among PSB treatments and with NP_SSP_K among uninoculated treatments. The soil K content was significantly higher in nine PSB treatments than other PSB treatments, NP_0_K, NP_TCP_K and NP_SSP_K. The highest available K was recorded for NP_TCP_K+*P*sp BIHB 756. The available Ca was significantly higher in three PSB treatments than other PSB treatments, NP_0_K, NP_TCP_K and NP_SSP_K.

## Discussion

The organic acid production during solubilization of inorganic phosphates by the efficient phosphate-solubilizing strains of *Pseudomonas trivialis*, *Pseudomonas poae, Pseudomonas fluorescens *and *Pseudomonas *spp., corroborated their involvement in phosphate solubilization [1,3,6]. Gluconic acid was the major organic acid produced as reported during phosphate solubilization by *Pseudomonas *sp. [16], *P. fluorescens *[17], *Azospirillum *spp. [18], *Citrobacter *sp. [19], and *Pseudomonas corrugata *[6]. The production of 2-ketogluconic, oxalic, malic, lactic, succinic, formic and citric acid in small quantities by *Pseudomonas *strains have also been reported during phosphate solubilization by *Arthrobacter ureafaciens*, *Arthrobacter *sp., *Bacillus coagulans*, *B. megaterium*, *Chryseobacterium *sp., *Citrobacter koseri*, *Delftia *sp., *Enterobacter intermedium*, *Pseudomonas fluorescens*, *Rhodococcus erythropolis *and *Serratia marcescens *[3,6,16,20,21]. None of *Pseudomonas *strains produced propionic acid unlike *Bacillus megaterium *strains during phosphate solubilization [3].

The results indicated that the quantity of organic acids produced differed with the nature of phosphate substrates and *Pseudomonas *strains (Tables 2, 3, 4, 5). The higher solubilization of TCP than URP, MRP and NCRP could possibly be due to the higher gluconic acid production in presence of TCP. The lower production of gluconic acid and lower TCP solubilization by *Pseudomonas *sp. BIHB 751 than other *Pseudomonas *strains substantiated the involvement of gluconic acid in solubilization of calcium-bound phosphates. Succinic acid also appeared contributing to TCP solubilization as it was produced by high TCP-solubilizing strains and not by low TCP-solubilizing *Pseudomonas *sp. BIHB 751 strain. The lack of oxalic acid production by efficient phosphate-solubilizing *Pseudomonas *strains signified non involvement of oxalic acid in TCP solubilization though this acid has been implicated besides citric, gluconic, lactic and succinic acids in phosphate solubilization in alkaline vertisols [20]. *Pseudomonas *sp. strain BIHB 751 producing the highest quantity of 2-ketogluconic acid but showing the lowest TCP and URP solubilization also differed from *Enterobacter intermedium *reported for the enhanced phosphate solubilization with increasing 2-ketogluconic acid production [21]. Likewise, no relationship could be ascertained between the quantity of organic acids produced and the solubilization of rock phosphates by *Pseudomonas *strains as the highest solubilization observed for NCRP among the rock phosphates was coupled to the lowest production of total organic acids (Tables 3, 4, 5). Previously also the quantities of solubilized phosphorus could not be correlated with the quantities of organic acids in the culture medium [22]. UPR, MRP and NCRP have fluorapatite structure with the highest substitution of phosphate with carbonate in NCRP [23]. The higher solubilization and lowered quantities of organic acids detected in the presence of NCRP could be due to the higher reactivity and greater diversion of organic acids in the neutralization of free carbonates in the solubilization of NCRP as compared to MRP and URP [23,24]. Likewise, the higher solubilization and higher production of organic acids in the presence of TCP could be attributed to its amorphous nature with simple structure and absence of any free carbonates as compared to the crystalline lattice structure of the rock phosphates [25].

Cluster analysis of organic acid profiles generated different groups revealing inter and intra-specific variation in the production of organic acids by *Pseudomonas *strains (Fig. 2). The strains clustered together and those standing outside the clusters or sub-clusters belonged to different *Pseudomonas *species characterized previously by 16S rRNA gene sequencing [8,9]. The strains standing outside the clusters differed qualitatively and/or quantitatively from other strains in the production of organic acids (Tables 2, 3, 4, 5). The results implied that *Pseudomonas *strains are independent of their genetic relatedness in their phosphate-solubilizing ability and organic acid production even under similar set of culture conditions. Phosphate solubilization is a complex phenomenon which depends on the nutritional, physiological and growth conditions of the culture [26].

The enhanced growth and higher N, P and K contents in maize with PSB treatments underlined the advantage of phosphate-solubilizing activity of microorganisms for plant growth promotion (Table 6 and 7). The increased growth and P uptake have been reported on PSB inoculations with *Pseudomonas *sp. and *Serratia marcescens *in maize [17], *Pseudomonas fluorescens *in peanut [27], *Bacillus circulans *in mungbean [28] and *Pseudomonas *sp. in wheat [29]. The TCP solubilization in soil by fluorescent *Pseudomonas *strains as evidenced by *in vitro *TCP solubilization, increased soil P availability and higher plant P content would be useful particularly in the cold deserts of Lahaul and Spiti where soil P deficiency is attributed mainly to the reaction of P with calcium carbonate and calcium sulphate forming insoluble di- and tricalcium phosphates. The rock phosphates recommended for acid soils are reportedly not effective in alkaline soils as P source for the crops [30]. The significantly higher plant growth and N, P, and K content in plant tissues and soil with some PSB treatments over NP_SSP_K might be due to the immobilization of applied P by native soil microbiota and physico-chemical reactions in the soil. The increased and continuous P availability in the soil promotes biological nitrogen fixation [27]. No correlation among TCP solubilization, production of organic acids and plant growth promotion could be established as the highest solubilization and plant growth promoting activity was observed for *P. trivialis *BIHB 745 not showing the highest organic acid production. However, the lowest organic acid production and plant growth promotion by *Pseudomonas *sp. BIHB 751 showing the lowest TCP solubilization suggested that phosphate solubilization is an important mechanism of plant growth promotion. *Pseudomonas *strains exhibiting high TCP solubilization *in vitro *differed significantly in enhancing the plant growth in the soil indicating interplay of some other growth factors besides phosphate-solubilization (Tables 2, 6, and 7). Apart from making P available to the plants, phosphate-solubilizing microorganisms improve plant health directly by the production of phytohormones [31]. *Pseudomonas *strains have been reported to vary in their ability for phytohormone production [32-34]. The bacterial strains also differ in utilizing root exudates in producing biologically active substances and root colonizing ability known to influence the plant growth-promoting action of rhizobacteria [35]. Plant-microbe interaction is a complex phenomenon with the interplay of several mechanisms and environmental factors.

The decrease in soil pH in PSB treatments indicated the production of organic acids by *Pseudomonas *strains as also reported for phosphate-solubilizing *Aspergillus niger *and *A. tubingensis *[36]. However, less pH decline in soil during plant growth promotion experiments than phosphate solubilization in culture medium could be due to the buffering nature of soil [20]. The inorganic acids and H^+ ^ions of microbial origin and H^+ ^ions released from the plant roots during ammonium assimilation are also reported to influence the soil pH [22,30,37]. The studies have shown potential for plant growth promotion by *P. trivialis *BIHB 745, *P. trivialis *BIHB 747, *Pseudomonas *sp. BIHB 756 and *P. poae *BIHB 808 in the presence of TCP as the phosphate source. The native phosphate-solubilizing and stress-tolerant *Pseudomonas *strains are expected to cohabitate as effective microbial inoculants with the crops grown in the cold deserts of Lahaul and Spiti.

## Conclusion

The present study revealed that the innate ability of organic acid production by *Pseudomonas *strains is independent of their genetic relatedness. Significant difference in plant growth promotion among the efficient phosphate-solubilizing *Pseudomonas *strains point at the need for selecting the potential strains based on plant growth promotion in the soils supplemented with insoluble phosphates for their targeted application. The PSB strains with high potential for TCP solubilization appear promising for application in the Ca-rich and P-deficit soils in the cold deserts of Lahaul and Spiti for which field studies are required.

## Authors' contributions

PV carried out the experiments on phosphate solubilization, organic acid profiling, plant growth promotion and chemical analyses, data analyses, and manuscript writing. AG contributed in experimental designing, interpretation of results, co-ordination and supervision of the experimental work, manuscript writing and editing.
